# Cartesian acquisition with PR-like sampling: applications to 3D contrast-enhanced MRA

**DOI:** 10.1186/1532-429X-11-S1-P279

**Published:** 2009-01-28

**Authors:** Stephen J Riederer, Clifton R Haider

**Affiliations:** Mayo Clinic, Rochester, MN USA

**Keywords:** Receiver Coil, Parallel Acquisition, View Sharing, Plantar Arch, Receiver Coil Array

## Introduction

View sharing and parallel acquisition are methods which can be used to improve the frame rate in time-resolved studies. In this work these methods are integrated and applied to contrast-enhanced MR angiography.

## Purpose

CAPR for "Cartesian Acquisition with Projection Reconstruction-like sampling" integrates Cartesian k-space sampling, 2D acceleration techniques, and view sharing to provide 3D time-resolved image sequences of contrast-enhanced MR angiograms. In this work we describe its development and application to multiple vascular territories.

## Methods

Prior to performing in vivo studies the CAPR technique was evaluated technically. Receiver coil arrays were developed having as many as 16 elements, routinely allowing 2D SENSE acceleration of 10 or higher, with overall acceleration of 15 using 2D homodyne reconstruction. For imaging the lower legs, elements placed anterior-posterior were designed for smaller depth of response than those placed left-right so as to limit loss of SNR in 2D parallel acquisition. The portrayal of the leading edge of contrast was tested using a computer-controlled bolus phantom. This was used to evaluate edge blurring as well as absolute accuracy of position and velocity. CAPR was used to image the vasculature of the brain and the extremities at 3.0 T. For intracranial vasculature the spatial resolution is 1 mm isotropic with image update times of 1–3 sec. For bilateral imaging of the lower legs, feet, and hands the parameters were adjusted for sub-mm isotropic resolution and frame times of 4–7 sec

## Results

Modified receiver coils as described can keep g-factors for the lower legs well under an absolute value of 1.5 even for accelerations as high as 8. The phantom studies show that CAPR can effectively freeze the motion of the advancing contrast bolus, with blur of the leading edge less than 10% of the distance moved from one time frame to the next. Image reconstruction is performed immediately, with the time series of MR angiograms available for display within two minutes after complete of the acquisition. In dozens of in vivo studies CAPR has been shown to consistently provide images of high spatial resolution, with temporal resolution clearly distinguishing arterial from venous phases and with 2D SENSE accelerations of 8 or higher. For the brain the method allows 1 mm imaging of the arterial vasculature with negligible venous signals. For the lower legs the technique depicts the vasculature at the level of third order branching, even in subjects with fast arterial-to-venous flow patterns. For the feet the method routinely depicts progressive filling of the tarsal and plantar arches.

## Conclusion

CAPR has been readily adaptable to all vascular areas studied thus far. It provides time-resolved 3D data sets with very high (1 mm isotropic) spatial resolution and which clearly portray arterial and venous enhancement patterns.

